# Grade IV Sacral Sore Treated with an Ointment Rich in PUFAs, Ceramides, and Antimicrobial Peptides

**DOI:** 10.1155/2022/4445055

**Published:** 2022-03-07

**Authors:** Marta Cassini, Irina Saretzky

**Affiliations:** University of Buenos Aires, Buenos Aires, Argentina

## Abstract

This report presents the case of a 57-year-old male patient with a history of hypertension, obesity, dyslipidemia, and coronary disease that after a prolonged postcoronary surgery hospitalization developed a sacral butterfly-shaped sore, with asymmetric involvement of the base of both buttocks, grade III on the left and grade IV on the right sides. The lesion was very painful and had a negative impact on the patient's sleep and mood. Following the initial surgical debridement and treatment with collagenase ointment, the wound showed delayed healing, an increase in necrotic tissue, and purulent discharge, requiring a second surgical debridement that revealed a deeper involvement of the wound. After a month with poor therapeutic response, it was decided to change the treatment to the application of gauzes embedded in an ointment rich in polyunsaturated fatty acids (PUFAs), ceramides, and antimicrobial peptides, Curefini® ointment. After 30 days of treatment, a 50% reduction of the initial wound diameter was obtained with a positive impact on the patient's quality of life, together with a reduction of incidental and spontaneous local pain resulting in better night rest and a return of appetite and strength. During the treatment course, the patient did not suffer any local or systemic infection in connection with the wound. After one month, the patient could discontinue the use of opioid analgesics. A 95% closure of the lesion was achieved in 45 days. This case highlights the therapeutic efficacy of the treatment of cavitated wounds with a product based on natural ingredients that helps reduce pain and promotes granulation and reepithelialization.

## 1. Introduction

Chronic wounds are defined as wounds nonresponsive to initial therapy or persistent in the face of appropriate care, with no significant progress towards healing in 30 days [1]. Chronic wounds have several distinctive features that affect their ability to heal in a timely manner. Alterations in cellular function and biochemical balances can keep a chronic wound “stuck” in the inflammatory, proliferative phase of healing. Cells in a chronic wound become senescent and do not respond to chemical messengers. Cytokines and growth factors, essential to the healing of the wound, become ineffective in senescent cells. Chronic wound fluid contains chronic inflammatory cells as a result of the prolonged inflammatory proliferative phase. The effect is dramatic. Apoptosis, the genetic code to normal cell death, is inhibited and delayed. There is defective extracellular matrix remodeling, failure in reepithelialization and wound-edge migration and an increase in senescent fibroblasts [2].

Pressure ulcers are considered chronic cutaneous wounds as the healing process is prolonged and slow, with a second-intention healing strategy. These lesions are usually located in the skin and underlying tissues on bony prominences and are caused by sustained pressure over time, deformation, friction, and rubbing [3].

Pressure ulcers represent a major problem in the health care system, with great epidemiologic, economic, and socio-family impact [4]. Approximately 65% of pressure ulcers originate in hospital stays, affecting mostly patients above 65 years of age and producing a negative impact on their nutritional and metabolic status and alteration in bowel movement, in mobility, cognitive, and perceptual capacity, and on the skin barrier [5].

Hospital-acquired pressure ulcers are considered as one of the healthcare quality indicators, and it is estimated that 55 to 77% of cases could be avoided [6]. Early detection of patients at risk of developing a pressure injury needs to be implemented from the time of admission. Age, incontinence, and body mass index (BMI) are well-established risk factors. Having a BMI <19 kg/square meter and age above 65 are the most significant risk factors [7, 8]. Incidence is lower in overweight (BMI 25–30 kg/square meter) and obese (BMI >30 kg/square meter) patients [9].

The Braden Scale assesses the risk of developing a pressure injury based on criteria related to activity, mobility, skin moisture, nutritional status, friction, and rubbing, as well as the ability to feel pain and discomfort in different parts of the body as a result of pressure [10, 11]. Charlson's Comorbidity Index permits the assessment of comorbidity factors related to the mortality risk of hospitalized patients [12]. Use of this index to predict the mortality of patients with pressure ulcers has shown moderate to high risk when comorbidities include vascular disease (myocardial infarction, congestive heart failure, peripheral vascular disease, and cerebrovascular disease), diabetes, renal disease, chronic pulmonary disease, and metastatic cancer [13].

Another important aspect in risk evaluation can be the categorization by related risk groups; this means clustering groups of surgical patients and nonsurgical patients during hospitalization, with the surgical group being the one that bears the highest risk [14]. Surgical patients are at higher risk of developing pressure ulcers due to factors such as longer immobilization, anesthesia and/or surgery, and preexisting medical conditions [15, 16]. In the United States, patients with hospital-acquired pressure ulcers imply longer hospital stays and higher indirect costs [17]. The prevalence of hospital-acquired pressure ulcers in surgical patients amounts to approximately 8.5% or higher, depending on the type and duration of the surgery [18]. Many hospitals use the Braden Scale to assess surgical patients' risk of developing pressure ulcers; this evaluation can be done one day before or after the surgery [19]. A low total value on the Braden Scale means a higher risk of developing pressure ulcers [20]. The Braden Scale has proven at least 70% sensitivity and specificity to identify the risk of developing pressure ulcers in hospitalized patients [21]. However, this scale does not include the abovementioned risk variables and comorbidities. Thus, it is suggested not to use it alone to predict pressure ulcer risk in surgical patients [22].

Once the pressure ulcer has formed, the selection of an appropriate therapeutic strategy will determine the time of evolution, the type of response of the injured tissue, related infectious complications, and the total cost of medication and related procedures.

In the treatment of cavitated wounds, different therapeutic options focus on stimulating granulation at the dermis level to achieve coverage of underlying structures such as muscles, bones, or tendons. Crevices and tunneling present in this type of lesion pose a therapeutic challenge as does the management of wound discharges and the prevention of site contamination by feces or urine, which require advanced care by staff skilled in the management of smart dressings and wound devices. The presence of odor, peri-ulcer inflammation, and local pain need to be considered when selecting a therapeutic strategy. Currently, calcium alginate is frequently used, as well as negative pressure systems or grafts for cavitated wounds. The choice will depend on the availability of financial and human resources and hospital infrastructure.

Advanced wound care, proper diagnostic understanding in each individual case, and management of the patient and of his/her family context are all necessary conditions to achieve good clinical results.

Insufficient availability of skilled human resources as well as the high cost of materials required for advanced care of wounds in developing countries calls for the development of easy-to-implement, reproducible, and economical strategies that can be supervised by telemedicine.

In this article, we present the clinical case of a cavitated, grade IV wound treated with gauzes embedded in an ointment based on natural components derived from cod liver oil, sunflower oil, sweet almond oil, and bee wax, showing the high capacity of the ointment to favor granulation with appropriate control of the inflammatory process, thus avoiding the build-up of bacterial colonies and providing quick relief of local pain. The article stresses that the ease of application of the product for advanced care of grade IV cavitated wounds permitted continuity of treatment in remote locations by nonspecialized personnel.

## 2. Case Presentation

We present a case of a 57-year-old male patient with a history of hypertension, dyslipidemia, obesity, and coronary disease that underwent double bypass surgery, with a 27-day-postop interment period during which he developed a sacral pressure ulcer with asymmetrical compromise on both sides of the intergluteal cleft, grade III and grade IV pressure ulcers on the left and right sides, respectively, according to the NUAP/EUAP classification [23].

At home, the patient felt weak, could barely move, and complained of intense local pain in the sacral area that disabled him to sit down or sleep well. The patient's wife incidentally discovered that a skin brownish lesion was present at both sides of the intergluteal fold, motivating a new medical consult with a plastic surgeon three days after. According to the patient's report, no advanced wound care treatment was established during his hospitalization and he lost 10 kg of weight.

**Figure 1:**
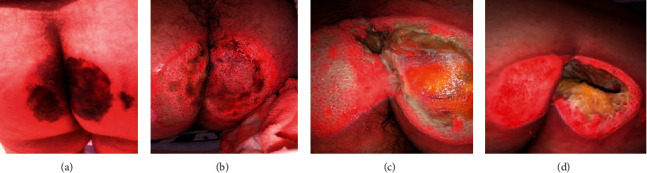
Wound progression before Curefini® treatment. (a) Day 1: the patient is discharged from hospital after 27 days. Chloramphenicol/collagen is initiated as local treatment. (b) Day 10: the first surgical debridement is performed. Chloramphenicol/collagenase local treatment continues. (c) Day 18: topical treatment with chloramphenicol/collagenase continues. (d) Day 20: the second surgical debridement is performed, initiated with iodoform gauze, antibiotics PO, and oral opioids.

The patient's photographs showed a sacral ulcer involving the intergluteal cleft in a butterfly shape, with necrotic tissue 10 cm diameter on each side. Involvement of the deep tissues was not evident at first sight, but the left side seemed to have more superficial involvement (Figure 1(a)). The treating physician decided to initiate autolytic debridement with chloramphenicol/collagenase ointment plus secondary gauze dressing with petrolatum, cleaning with polyhexanide solution twice daily.

After 10 days of evolution, the first surgical toilette was performed with debridement of necrotized tissue; no swabs or culture specimens were collected. An ulcerative granulation bed was observed in the deep dermis with active borders and some brownish red areas on both sides. No local erythema, temperature, or purulent discharge was observed (Figure 1(b)). The patient received prophylactic antibiotic therapy with ciprofloxacin 500 mg PO bid for five days and continued with chloramphenicol/collagenase treatment at home.

After 18 days of chloramphenicol/collagenase local treatment, an ulcerative bed was involving sectors up to the hypodermis with fatty tissue exposure and cavitation at the right median and paramedian sacral level, reaching the muscular plane with no evident bone exposure (Figure 1(c)). Neither the probe to bone nor the lumbosacral IRM was performed. A fetid, purulent discharge was found on the gauze. No fever or shivering was reported. It was decided to schedule a new surgical toilette which was performed on day 20.

On day 20, a second surgical debridement was performed. Physical examination showed a left-side grade III pressure sore with granulation tissue at the wound bed and active reepithelialization borders; on the right side, a cavitated grade IV sore was observed, with hypodermis fat exposure, increased depth of the cavity with no bone exposure, and negative probe to bone (Figure 1(d)).

Following the debridement, according to the patient's medical report, no soft tissue culture specimens were collected and empiric systemic antibiotic treatment was indicated: amoxicillin/clavulanate 875/125 mg PO bid for ten days and local treatment with iodoform gauze dressings inside the cavity, with the indication to change every 48 hours. The patient complained of an increase in local pain and received tramadol 50 mg PO every 8 hours.

On day 30, the treating physician proposed a skin graft; however, the patient decided to get a second opinion from a wound care specialist. At this time point, the authors contacted the patient for the first time. During the interview, the patient was said to be concerned about the evolution of the wound, was said to be unwilling to have a surgical procedure and later hospitalization since his home is located 500 km away from the hospital, and had reported continuous pain and impossibility to sit or find a good position to sleep and regarding his mood, he seemed to be more depressed.

As independent wound care specialists, we suggested the patient and his family a new local treatment consisting of gauzes embedded in an ointment made of cod liver oil, sunflower seed oil, sweet almonds, virgin beeswax, and vitamins A and D, Curefini® [24,25]. One month after being discharged from the Coronary Unit, the patient started topical treatment with a sterile gauze lubricated with Curefini®, filling the cavity. The surface of both buttocks was covered with the sterile gauze embedded with Curefini®, with the indication to change it every 12 hours.

**Figure 2:**
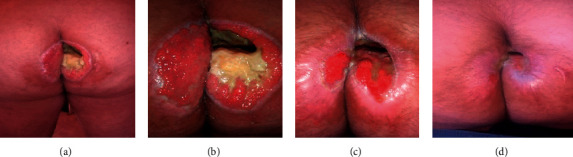
Wound progression with Curefini® treatment. (a) Day 42: after 12 days of Curefini® local treatment. (b) Day 60: after 30 days of Curefini®. (c) Day 75: after 45 days of Curefini® local treatment. (d) Follow-up after three months.

On day 42, after 12 days of topical treatment with natural ointment, pain improved significantly, oral opioids were tapered until total discontinuation, and the patient tolerated the sitting position for brief periods. After Curefini® local treatment was established, the granulation and reepithelialization processes were reactivated and hypodermis fat tissue was covered with new skin (Figure 2(a)).

On day 60, after 30 days of treatment with Curefini®, a 50% reduction in transverse wound diameter was observed, along with granulation tissue in the cavity bed and advanced epithelialization border (Figure 2(b)).

On day 75 after discharge from the hospital, with a 45-day-continuous period of Curefini® treatment, 95% closure of the lesion was achieved and a large crater ulcer was replaced by a transverse fissure in the right intergluteal fold of only 4 cm in depth, which continued to be treated locally with a gauze impregnated with the ointment (Figure 2(c)).

At this point, the patient starts to walk without difficulty or pain, maintains a correct diet with normal bowel movements, has a restorative sleep, and shows a tranquil mood.

Control after 3 months of Curefini® treatment showed the cavitation was completely filled, although a certain degree of scar retraction is observed in the surrounding tissue with some hypertrophy. No remnant fistula trajectory or tunnel is observed.

## 3. Discussion

During the last decade, the therapeutic approach to cavitated pressure ulcers has made great progress with the advancement of new technologies such as negative pressure, which promotes the filling of deep wounds [26]. Negative pressure cannot be applied in the presence of cancer tissue in the wound, to untreated osteomyelitis, in wounds with organ or vessel exposure, or to areas with poor blood flow. In some trials, its use in infected grade IV sacral ulcers has proved advantageous in terms of the recovery time as compared to conventional treatments, achieving recovery time duration of approximately 6 weeks [27]. However, in developing countries, this type of device continues to have a high cost, not only because of the elements required but also because of the skilled staff required for correct application and removal.

Other options are flaps and grafts close to the anal region, but these may lead to complications such as infection and necrosis. Usually, the sacral and perianal regions create difficulties for the adjustment and fixation of any device. Combined techniques and strategies, such as flaps plus a negative pressure system, tend to work well, although they also imply greater therapeutic demands [28].

A low-cost option for the treatment of cavitated wounds is calcium alginate, alone or in combination with micronized silver, with high absorptive and hemostatic capacity and some bactericidal properties. Calcium alginate may be applied in rope dressings that are easily introduced in tunneled wounds and can easily integrate naturally into the tissue. The dressings can be changed every 72 hours. When this product dissolves, it releases a gel-type discharge from the wound bed with a special smell that may be mistaken with infection. It requires a secondary dressing [29].

In the case of our patient, the treatment strategy was conditioned by previous established therapeutic actions, distance from the medical center, and economic limitations. On the one hand, during a 20-day period of time since patient discharge, no soft tissue culture specimens were collected and empiric broad-spectrum antibiotic treatment was indicated. We assume that this choice was aimed at preventing and/or treating a possible but not proven soft tissue local infection. In such cases, wound irrigation and debridement of necrotic tissue are the most important factors in the prevention of infection and can substantially decrease the incidence of invasive wound infection. Antibiotic prophylaxis is not generally recommended (recommendation 1C). However, in patients with systemic signs of infection, compromised immune status, severe comorbidities, associated severe cellulitis, and severe and deep wounds, a broad-spectrum antibiotic effective against aerobic and anaerobic organisms is always required (recommendation 1C) [30]. According to the medical history, the patient did not show any signs of systemic compromise like fever or shivering, but bad odor and purulent discharge were observed locally. No visible bone was found in the physical exam, the probe to bone was negative, and the C-reactive protein alteration or leukocytosis ruled out osteomyelitis. Nevertheless, we think that a magnetic resonance imaging (MRI) scan would have helped to better understand the complete structures compromised beneath and that a deep tissue biopsy and culture should have been collected. Due to the lack of resources and the large distance from a high-complexity health center, those were not performed [31]. On the other hand, intense local pain led the patient to refuse any kind of aggressive treatment at the wound site. Oral opioids were indicated by the plastic surgeon.

Pain associated with chronic wounds has been postulated to be related to the stimulation of nociceptors by complex inflammatory environments [32]. Molecular studies investigating keratinocyte biology and wound healing have shown that functionally active *μ*-opiate receptors are present on human keratinocytes [33]. The activation of these receptors by *μ*-opiate receptor agonist *β*-endorphin in cultured keratinocytes results in upregulation of type II transforming growth factor-*β* (TGF-*β*) receptor and cytokeratin 16 (CK16) [34]. The type II TGF-*β* receptor plays an important role in wound healing, and it is expressed in regenerating epithelial cells in acute wounds and at the margin of chronic wounds. CK16 is a filament protein that is not expressed in normal skin but appears in the suprabasal compartment of the epidermis during wound healing. Additional experiments using cultured keratinocytes show that CK16 response can be blocked by incubation together with the *μ*-opiate receptor antagonist naltrexone [34, 35]. These findings suggest that, hypothetically, the clinical use of opioids in patients with chronic wounds might be beneficial in upregulating molecular pathways contributing to wound healing and may be associated with improved clinical outcomes. Other studies have suggested that opioid use may negatively impact wound healing by reducing immune activation and impacting tissue oxygenation and angiogenesis [36, 37]. In contrast, untreated pain may affect tissue perfusion and oxygenation [38, 39]. The Wound Etiology and Healing Study (WE-HEAL) (IRB 041408, NCT 03152078) investigates the relationship between patient-reported pain, opioid exposure, and wound outcome in the clinical care of a longitudinal cohort of patients with chronic wounds. This study corroborates at a cellular and molecular level that opioid exposure may impact keratinocyte biology and wound healing, resulting in delayed wound healing in patients with chronic wounds. It also concludes that opioid exposure was a strong predictor of wound size and that patients who received opioids above 10 mg per day had slower rates of healing than those with no exposure or dosing less than 10 mg per day [40].

Since the patient completed a 20-day empiric antibiotic therapy, the ulcer appeared to be clean after the last surgical debridement, and the patient showed no systemic compromise, it was decided to use a gentile wound care strategy, with a less-aggressive debridement technique aimed at reducing incidental pain. It was decided to use a natural ingredient ointment embedded in sterile gauzes. Its main components are polyunsaturated fatty acids (PUFAs) from cod liver oil, sunflower oil, sweet almond oil, antimicrobial peptides from virgin beeswax, and vitamins A and D. A clear knowledge of the action of its main components in the healing process and the ease of application, added to the low cost, led to the selection of this product, also taking into consideration the patient's home distance from the hospital for regular controls that were conducted by videos and photos sent by his wife and weekly telephone calls. Curefini® ointment is a formula enriched with polyunsaturated fatty acids (PUFAs), omega 3, and omega 6, contained in its pure source oils. PUFAs reduce the local inflammatory process in the wound by inhibiting the production of inflammatory eicosanoids and competitive inhibition of the formation of arachidonic acid. PUFAs also have antibacterial action [41]. Sunflower seed oil contains large amounts of linoleic acid, which has anti-inflammatory and antibacterial properties and promotes restoration of the skin barrier [42]. Sweet almond oil is restorative to the skin barrier and has antipruritic properties [43]. Beeswax is known for its healing, antibacterial, and anti-inflammatory properties [44, 45]. The combination of cod liver oil and medicinal beeswax has been successfully used in wound treatment in veterinary medicine [46, 47]. Curefini® has been previously tested on a porcine model for second-degree burns and showed the ability to control the inflammatory process and promote the development of a more-resistant skin layer in the affected area [25].

Vitamins A and D participate in normal skin function and collaborate as antioxidant cofactors.

Vitamin D is a pleiotropic molecule that has widespread effects not only on calcium homeostasis but also on cellular differentiation, proliferation, and immune responsiveness [48, 49]. 1,25-dihydroxyvitamin D (1, 25D3), the active form of vitamin D, has been widely studied for its immunomodulatory properties and is known to suppress inflammation in a variety of tissues, largely through its influence on antigen-presenting cell differentiation, lymphocyte proliferation, innate immune receptor signaling, and cytokine and chemokine expression [50–52]. In addition to its beneficial effects during inflammatory events, vitamin D also induces the expression of antimicrobial peptides [53–55], potentially providing enhanced protection during infection and wound healing.

Vitamin A is a family of retinoids that are available as preformed vitamin A (retinol, retinaldehyde, and retinoic acid) or as pro-vitamin A (carotenoids). Vitamin A is an essential fat-soluble micronutrient that cannot be synthesized by the human body and must be obtained from the diet. It is available in eggs, fish oils, liver, dairy products, yellow and orange fruits, and dark-green leafy vegetables. Vitamin A promotes cell mitosis and increases epithelial thickness, the number of Langerhans cells, and glycosaminoglycan synthesis in the skin. Studies have shown that vitamin A functions as a hormone, altering the activity of epithelial cells, melanocytes, fibroblasts, and endothelial cells through its action on the family of retinoic acid receptors (RARs) [56, 57]. It maintains the health of epithelial cells on the surface of the skin and the mucous membranes. It stimulates the immune system, maintains mucosal and epithelial integrity, and increases collagen and extracellular matrix formation, fibroplasia, and synthesis of collagen, glycoprotein, and proteoglycan [58–60]. It enhances the epithelialization of cell membranes, the rate of collagen synthesis, and cross-linking of newly formed collagen and antagonizes the inhibitory effects of glucocorticoids on cell membranes [61]. Vitamin A requirements are exceedingly in demand during serious injury or stress. Burns, fractures, and even elective surgery can lead to decreased serum levels of vitamin A, retinol-binding protein (RBP), retinyl esters, and *β* carotene. Also, liver storage of vitamin A can be depleted by large doses of corticosteroids [62]. Vitamin A is known for its ability to stimulate epithelial growth, fibroblasts, and granulation tissue. Many dermatologic conditions have been treated using topical formulas. Moreover, early in the inflammatory phase, vitamin A facilitates epithelial cell differentiation in open wounds by increasing the number of monocytes and macrophages and thus acts as an anti-inflammatory agent [63]. It seems that vitamin A has the potential to be provided topically or orally. Wounds can be acute (such as the trauma of lacerations, crush wounds, or burns) or they can be chronic (such as diabetic foot ulcerations). Vitamin A can promote and enhance various aspects of wound healing via the stimulation of angiogenesis, collagen synthesis, epithelialization, and fibroplasia. Local (topical) and systemic supplementation with vitamin A has been proven to increase dermal collagen deposition [64]. Topical administration of vitamin A has numerous dermatology indications, but its application directly on wounds has not been established as a treatment modality, despite some data suggesting it helps with diabetic foot ulcers and venous leg ulcers [65, 66].

Curefini® is an OTC product approved by the FDA and is currently in use in pediatric patients with recessive dystrophic epidermolysis bullosa [46, 67]. Its use in adults with second-degree burns has recently been reported with promising results regarding cosmetic and functional achievements [47, 68]. Curefini's main components are petrolatum, sunflower oil, cod liver oil, and sweet almond oil. Medical beeswax is present in the formula in a lower proportion than the previously mentioned oils. Curefini PUFA's component gives anti-inflammatory properties that facilitate a controlled wound inflammatory environment, enhance reepithelialization and granulation processes, and allow for autolytic debridement and gentile mechanical detritus removal t on each gauze change. Antibacterial peptides present in the beeswax component bring additional antibacterial properties [69–72]. Regarding the anti-inflammatory activity of Curefini®, a remarkable clinical aspect is that patients with second-degree burns report immediate pain reduction [68] with its use. Although our patient had a chronic wound, the Curefini® anti-inflammatory action allowed us to taper opioid therapy with no need for further pain relief replacement.

There are commercial products that share some of the components present in Curefini® ointment. For example, L-Mesitran contains medical-grade honey (MGH) as the main active ingredient and shows promising results in treating severe wounds in pediatric patients [72]. This honey-based wound care product contains 48% medical-grade honey, hypoallergenic lanolin, vitamin C, vitamin E, zinc oxide, and essential oils. Several studies have demonstrated that the supplements in L-Mesitran enhance the antimicrobial properties of its raw honey. In the pediatric case series presented by Smaropoulos [72], we note similar rates of response in wound healing reactivation and no infectious complications as with Curefini®. Both in adult and pediatric patients, the Curefini® restorative action relies on its preponderant PUFA composition provided by fish oil, sweet almond oil, and sunflower oil. Additionally, it has antimicrobial activity thanks to the bee wax component, but not as the main active compound. Vitamins A and D, as explained before, contribute with an excellent restorative and antioxidant action.

No comparison trials of Curefini® vs. other related formulas have been done. However, this case report highlights that Curefini® can be a reliable treatment for grade IV sacral pressure ulcers in adult patients.

## 4. Conclusion

This case highlights the therapeutic efficacy of treatment with a natural ingredient-based product on cavitated wounds that helps to reduce pain and promote granulation and reepithelialization of the skin. We remark that despite the location of a cavitated sacral wound near the anus and patient depositions, no local or systemic infectious events were detected. Its use should be considered a treatment of choice in developing countries where the distance from the hospital, economic limitations, and lack of wound care specialists are key conditions for a successful outcome.

## Data Availability

Data are available on request to the authors Marta Cassini and Irina Saretzky (irinasares@yahoo.com.ar and mscassini44@gmail.com).
